# The photophysics of naphthalene dimers controlled by sulfur bridge oxidation†
†Electronic supplementary information (ESI) available. See DOI: 10.1039/c7sc01285c



**DOI:** 10.1039/c7sc01285c

**Published:** 2017-04-24

**Authors:** Clàudia Climent, Mario Barbatti, Michael O. Wolf, Christopher J. Bardeen, David Casanova

**Affiliations:** a Departament de Ciència de Materials i Química Física , Institut de Química Teòrica i Computacional (IQTCUB) , Universitat de Barcelona , Martí i Franquès 1-11 , Barcelona 08028 , Spain; b Aix Marseille Univ , CNRS , ICR , Marseille , France; c Department of Chemistry , University of British Columbia , 2036 Main Mall , Vancouver , BC V6T 1Z1 , Canada; d Department of Chemistry , University of California Riverside , 501 Big Springs Road , Riverside , California 92521 , USA; e Kimika Facultatea , Euskal Herriko Unibertsitatea (UPV/EHU) , Donostia International Physics Center , Paseo Manuel de Lardizabal, 4 , Donostia 20018 , Spain . Email: david.casanova@ehu.eus; f IKERBASQUE , Basque Foundation for Science , Bilbao 48013 , Spain

## Abstract

In this study we investigate in detail the photophysics of naphthalene dimers covalently linked by a sulfur atom. We explore and rationalize how the oxidation state of the sulfur-bridging atom directly influences the photoluminescence of the dimer by enhancing or depriving its radiative and non-radiative relaxation pathways. In particular, we discuss how oxidation controls the amount of electronic transfer between the naphthalene moieties and the participation of the SO_*n*_ bridge in the low-lying electronic transitions. We identify the sulfur electron lone-pairs as crucial actors in the non-radiative decay of the excited sulfide and sulfoxide dimers, which are predicted to proceed *via* a conical intersection (CI). Concretely, two types of CI have been identified for these dimers, which are associated with the photo-induced pyramidal inversion and reverse fragmentation mechanisms found in aryl sulfoxide dimers. The obtained results and conclusions are general enough to be extrapolated to other sulfur-bridged conjugated dimers, therefore proportionating novel strategies in the design of strongly photoluminescent organic molecules with controlled charge transfer.

## Abstract 2

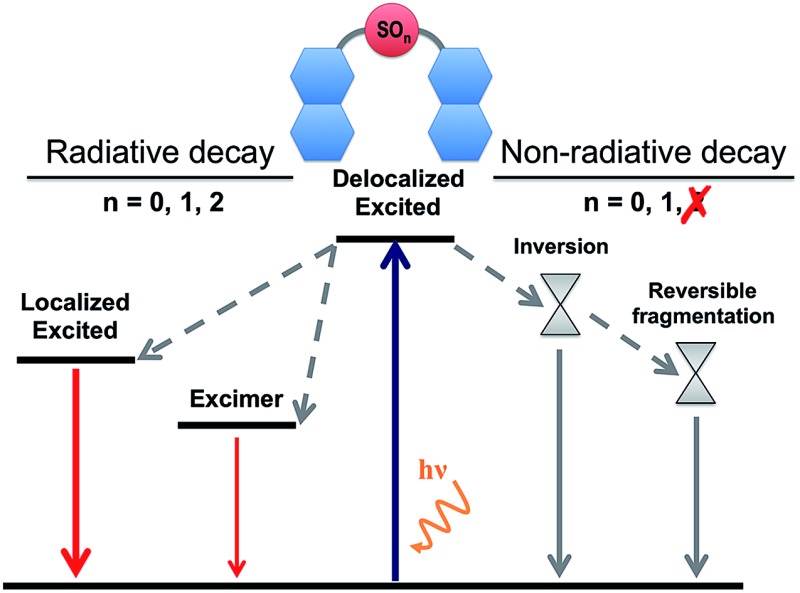
Oxidation state of bridge controls the deactivation mechanism of naphthalene dimers.

## 1. Introduction

Organic photovoltaics (OPVs) represent a very promising alternative in the conversion of solar energy to electricity. Although OPVs have the potential to provide electricity at a lower cost than the first and second generations of solar technology, the current record efficiencies (∼13%)^1^ are still far too low to compete with the performance of silicon panels^2^ and other non-fossil energy sources. Organic solar cells present numerous advantages, *i.e.* an abundance of materials, well-developed organic chemistry for their synthesis, available chemical strategies to tune their properties and they can be produced as thin, flexible and light modules that can be easily manufactured at room temperature. However, in order for OPV technology to compete with other energy sources some fundamental obstacles need to be overcome. In particular, OPV technologies exhibit short device lifetimes and low dielectric constants, resulting in low energy conversion efficiency. One of the fundamental issues at the microscopic level is the generation of separated charges from the optical exciton. High exciton binding energies result in energy losses at the cell heterojunction that induce rather low open-circuit voltages (*V*_OC_). A promising and sophisticated strategy to increase *V*_OC_ in OPVs is the use of symmetric molecular electron acceptors, such as covalent dimers of organic chromophores (bichromophores) that are able to undergo symmetry-breaking charge transfer (SBCT).^3–5^ In SBCT, the initial excitation generated by photoabsorption relaxes to an intramolecular charge transfer state that breaks the molecular symmetry. Then, electron CT at the donor/acceptor interface leads to an oxidized donor and a reduced acceptor separated by a neutral chromophore, preventing fast charge recombination. Organic bichromophores have also been proposed as highly emissive molecules to use in organic light emitting devices (OLEDs)^6,7^ as an alternative to large aromatic molecules. In addition to strong photoluminescence (PL), optimal molecular systems to be used in OLEDs must allow intermolecular CT.^8^

The range of applicability of bichromophores is expected to be related to the nature of the bridged monomers and their interactions. The electronic coupling between the conjugated moieties depends on the geometry and electronic structure of the covalent linker, and understanding the parameters that ultimately control and determine such interactions becomes critical for the design of molecular systems with the desired characteristics. Recently, it was shown that the covalent linkage between conjugated chromophores *via* a sulfur bridge has a large impact on the fluorescence efficiencies of the parent chromophores, with a large increase in the PL yield observed upon the oxidation of the bridging sulfur atom, indicating a clear strategy in the search of strong molecular emitters.^9^ This trend was later scrutinized in the case of terthiophene dimers.^10^ The study concluded that rapid intersystem crossing (ISC) to the triplet state manifold is the main deactivation process limiting the fluorescence quantum yield, as observed in pristine terthiophene.^11,12^ The ISC efficiency is reduced in the presence of intramolecular charge transfer (CT), which can be tuned by the oxidation state of the bridging sulfur group. The electron lone-pairs on the sulfur atom screen the electronic interactions between the two chromophores, decreasing the CT and allowing efficient ISC for the sulfide and sulfoxide dimers, resulting in a larger PL efficiency for the sulfone bridge with no electron lone-pairs on S.

**Scheme 1:**
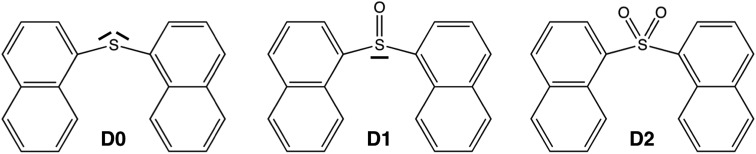
Molecular representation of the **Dn** dimers. The sulfur electron lone pairs are explicitly indicated.

Despite the much less efficient ISC expected for naphthalene due to the molecular planarity and lack of sulfur atoms, the PL in SO_*n*_ bridged naphthalene dimers exhibits the same trend as that found in the terthiophene analogues. Hence, in order to understand the origin of such behavior and to further explore the validity of the lone-pair screening concept, the present study features the photophysical properties of naphthalene covalent dimers linked through an SO_*n*_ bridge, where *n* = 0, 1 and 2. We label these three molecules as **D0** (sulfide), **D1** (sulfoxide) and **D2** (sulfone) throughout the paper (Scheme 1).

Our study is organized as follows. First, we discuss the relative stability of the structural conformers of the **Dn** dimers and their potential interconversion paths. Then, we explore the nature of the low-lying singlet states of the low-energy conformers and the structural and electronic properties of the local minima on the excited state energy surface. Finally, we discuss the availability of non-radiative decay pathways from the lowest excited singlet to the ground state.

## 2. Computational details

Electronic structure calculations for the ground and excited states were performed within the framework of density functional theory (DFT)^13,14^ and its time-dependent version (TDDFT),^15,16^ respectively. To take into account the weak interactions and important electronic redistribution between the naphthalene moieties and the SO_*n*_ bridge upon photoexcitation, the ωB97X-D functional^17^ was used together with the 6-31+G(d) basis set.^18–20^ Investigations on the dependence of the energy functional and basis set can be found as ESI (Tables S1 and S2†). Dichloromethane (DCM) solvent effects were taken into account with the polarizable continuum model using the integral equation formalism variant (IEFPCM).^21,22^ Critical points on the ground state potential energy surface (PES) were optimized with no restrictions and characterized within the harmonic approximation. The simulated emission spectra were calculated by convolution of the Gaussian functions (half-bandwidth of 2500 cm^–1^) centered at the computed vertical emission energies of all excited state minima and were averaged according to the ground or excited state Boltzmann populations (based on the relative electronic energies). Computation of the diabatic states was performed by means of the Edmiston–Ruedenberg localization scheme.^23^ Energy crossing points and derivative couplings between S_0_/S_1_ were computed at the spin-flip DFT (SF-DFT) approximation^24^ with the BHHLYP functional.^25,26^

All calculations were performed with the Gaussian09 package, revisions B01 and D01,^27^ and the Q-Chem program.^28^

## 3. Results and discussion

### 3.1. Thermal conformers

**Fig. 1:**
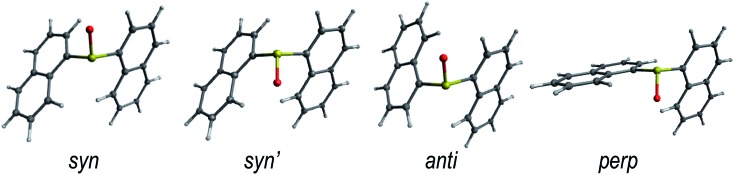
Lowest energy conformers for the ground state of the **D1** dimer in DCM solution. The low energy conformers of **D0** and **D2** are shown in Fig. S1.†

The rotation around the S-naphthalene bonds in the **Dn** dimers results in different structural conformers, which are local minima on the ground state PES (Fig. 1 and S1†).

The energetically lowest forms of **D0** and **D1** in solution correspond to the *syn*- and *anti*-arrangements of the naphthalene units with *syn* being slightly lower in energy (Table 1). As a result, it is reasonable to expect both conformers to be present in the solution as indicated by their Boltzmann fractions. On the other hand, **D2** shows a clear preference for the *anti* conformer, which is expected to be the main form found in DCM solution.

**Table 1:** The relative energies Δ*E* (in kcal mol^–1^), relative Boltzmann populations at *T* = 298 K (Pop. in%) and Mulliken charges (*q*) on the S and O atoms, and on the naphthalene moieties for the ground state optimized structures of the lowest energy conformers (conf.) of the **D0**, **D1** and **D2** dimers in DCM

Dimer	Conf.	Δ*E*	Pop.	*q* (S)	*q* (O_*n*_)	*q* (Naph_2_)
**D0**	*syn*	0.0	74	0.00	—	0.00
	*anti*	0.7	23	0.20	—	–0.20
	*anti*′	1.8	3	–0.04	—	0.04
**D1**	*syn*	0.0	69	0.68	–0.69	0.01
	*syn*′	3.5	<1	0.48	–0.69	0.21
	*anti*	0.5	30	0.68	–0.71	0.03
	*perp*	2.8	<1	0.54	–0.69	0.14
**D2**	*syn*	2.5	1	0.18	–0.97	0.79
	*anti*	0.0	94	0.27	–0.95	0.68
	*perp*	1.8	5	0.92	–0.99	0.08

It is worth noting that while **D0** only shows minor net charges in any of the fragments, *i.e.* the two naphthalenes and sulfur bridge in its *anti* conformer, the polarity of the S–O bond in the sulfoxide and sulfone bridges induces an electronic distribution towards the more electronegative O atoms (Table 1). Charge polarization in **D1** basically affects the SO linker, with the S and O atoms carrying positive and negative charges, respectively. The presence of the two O atoms in **D2** is able to pull considerable electron density from the two naphthalene units in the *syn*- and *anti*-conformers, resulting in a positive net charge on each chromophore. The different behavior observed for the *perp* form is related to the orthogonal orientation between naphthalene units, which results in asymmetrically charged chromophores.

In addition to identifying and characterizing the most stable conformations of the naphthalene dimers, it is also important to quantify the barriers for their interconversion. The structural transformation between the low energy conformers of each dimer can be achieved by the torsion of one naphthalene moiety with respect to the other one. The ground state energy profiles of these mechanisms are shown in Fig. S2–S4.† The computed barriers for the molecular torsion between the low-lying conformers are in the range of 2–6 kcal mol^–1^.

**Fig. 2:**
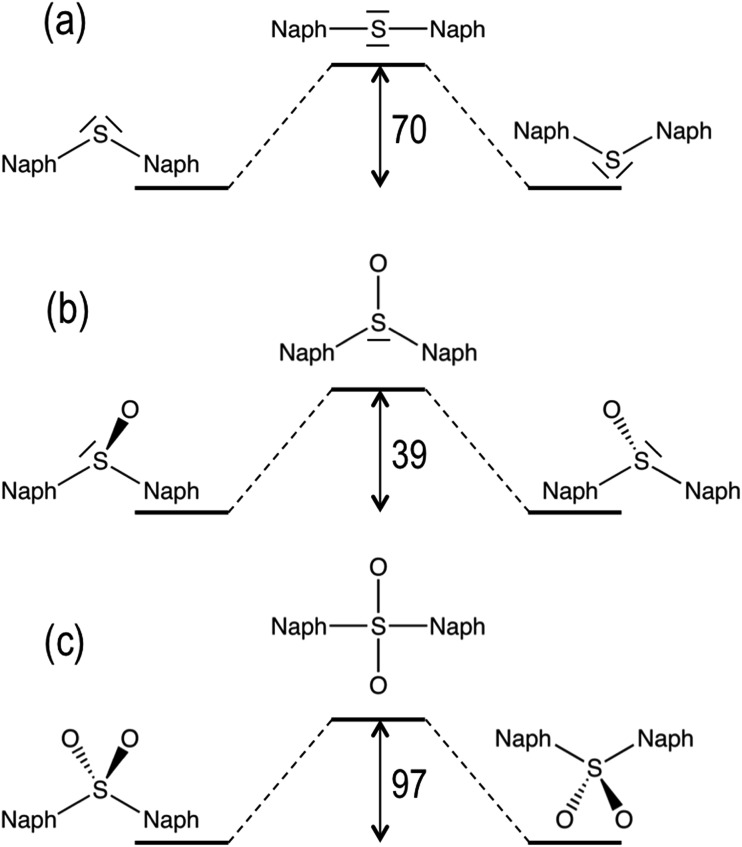
Ground state transition energy barriers (in kcal mol^–1^) for the structural inversion of **D0** (a), **D1** (b) and **D2** (c). The molecular representations are only meant to indicate the main differences between the S_0_ and TS geometries. The nature of the S–O bond (single or double bond) has been omitted for clarity.

In addition to molecular torsion, the conversion between the conformers of **D1** can be achieved by pyramidal inversion of the S atom (Fig. 2). The transition states for the inversion of the *syn*- and *anti*-conformers exhibit a planar geometry around the sulfur atom with a naph-S-naph angle close to 120°, *i.e.* corresponding to a trigonal planar geometry, and much larger than the ground state angle (close to the tetrahedral angle). The computed inversion barriers for the *syn* → *syn*′ and *anti* → *anti* conformational pathways are 38.4 and 38.6 kcal mol^–1^, respectively, in quantitative agreement with the computational estimations of the pyramidalization barrier for H_2_SO, DMSO^29^ and related sulfoxide heterodimers.^30^ Hence, thermal pyramidal inversion of the sulfoxide dimer is expected to be very slow, as previously observed for the racemization of aryl sulfoxides.^31,32^ Analogous to the pyramidal inversion observed in **D1**, the *syn*- and *anti*-conformers of the **D2** dimer may interconvert by a tetrahedral inversion through the planarization of the sulfone center through a square planar transition state. Our calculations indicate that this geometry is very high in energy (97 kcal mol^–1^ with respect to the ground state *anti*-conformer), and hence thermal interconversion of the sulfone-bridged naphthalene dimer *via* planarization can be completely disregarded. In spite of the lack of oxygen atoms in the **D0** bridge, it can also experience a similar inversion of the molecular structure by increasing the naph-S-naph angle at the bridge from the tetrahedral value (105°) in the *syn* and *anti*-ground state minima to a linear C–S–C disposition. Again, the computational estimation of the energy barrier for the structural inversion of **D0** is too high (70 kcal mol^–1^) to be thermally available, which is in very good agreement with the linearization energy estimated for H_2_S.^33^

### 3.2. Photoabsorption

The computed vertical excitation energies from the ground state to the lowest excited singlet state of the **Dn** dimers are rather close to each other regardless of the oxidation state of the sulfur bridge and lie within the range of 4.2–4.4 eV, in fairly good agreement with the experimental absorption maxima measured in DCM solution (Table 2). Moreover, the transition energies and oscillator strengths show small variations between the different conformers.

**Table 2:** The vertical transition energies Δ*E* (in eV), oscillator strengths (*f*), electronic characters (in%) LE (on the naphthalene fragments), CT (between the naphthalene moieties) and CT_B_ (from the SO_*n*_ bridge to the naphthalenes) and electronic couplings between the lowest LE, CT and CT_B_ diabatic states (in meV) for the lowest excited singlet of the most stable conformers of the **D0**, **D1** and **D2** dimers computed at the ωB97X-D/6-31+G(d) level

Dimer	Conf.	Δ*E* ^*a*^	*f*	LE	CT	CT_B_	LE/CT	LE/CT_B_
**D0**	*syn*	4.24	0.335	37	14	49	102	509
	*anti*	4.31	0.326	59	5	36	65	244
**D1**	*syn*	4.41	0.301	84	1	15	129	200
	*anti*	4.43	0.312	91	1	8	126	195
**D2**	*anti*	4.42	0.272	96	4	0	162	N/A

^*a*^Experimental absorption maxima: 4.11 eV (**D0**), 4.19 eV (**D1**) and 4.16 eV (**D2**).^9^

**Fig. 3:**
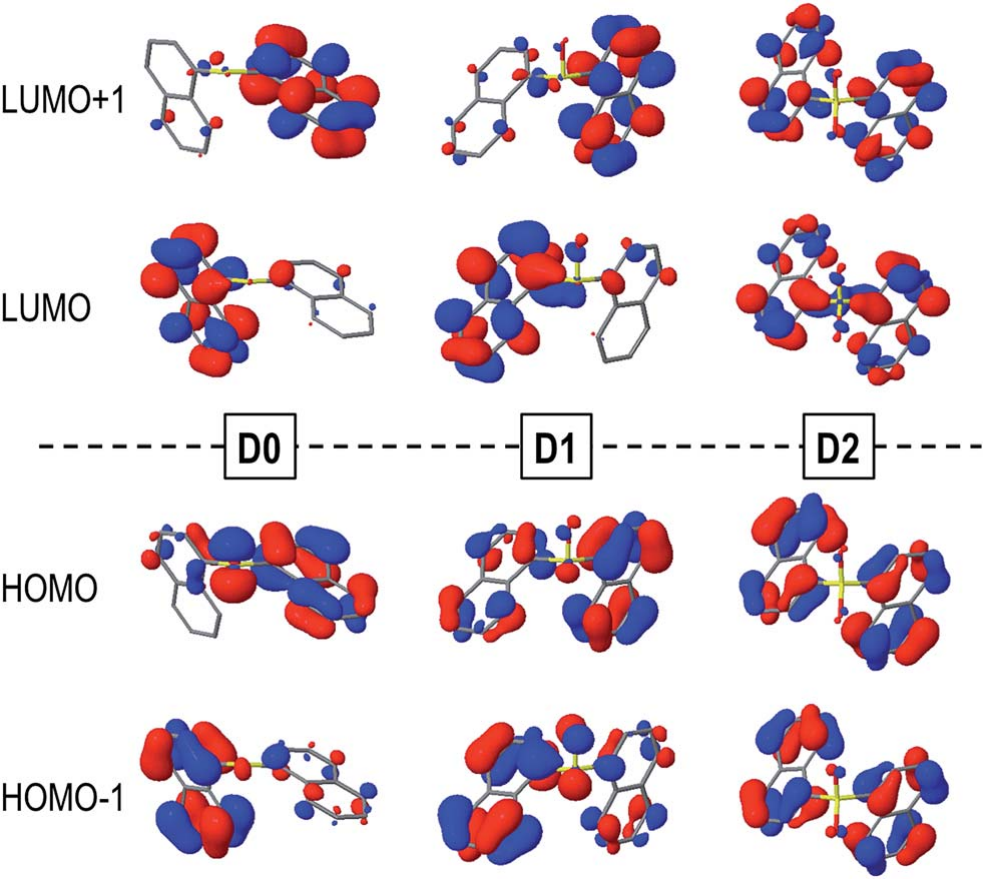
HOMOs (bottom) and LUMOs (top) of the *syn*-**D0** (left), *syn*-**D1** (middle) and *anti*-**D2** (right) dimers computed in DCM solution.

The main contribution to the lowest electronic transition from the most stable conformations (*syn* and *anti*) of the naphthalene dimers corresponds to a single electron promotion from the two highest occupied molecular orbitals (HOMO and HOMO–1) to the two lowest unoccupied molecular orbitals (LUMO and LUMO+1). The frontier orbitals are mostly delocalized over the two naphthalene moieties with some contribution from the SO_*n*_ bridge, mainly for the HOMOs of the **D0** and **D1** molecules (Fig. 3).

Despite the similarities between excitation energies and oscillation strengths of the S_0_ → S_1_ transition in the **D0**, **D1** and **D2** dimers, detailed electronic structure analysis brings to light the significant differences in the nature of the transition upon oxidation of the bridge (Table 2). Decomposition of the electronic transition in terms of diabatic states corresponding to the local excitations (LE) on the naphthalene moieties, charge transfer excitations between the aryl fragments (CT) and electronic transition from the bridge to the naphthalene chromophores (CT_B_) highlight the important differences in the nature of the vertical excitation upon oxidation of the sulfur atom linker (Table 2). In general, the main contribution corresponds to the π → π* excitations on both naphthalene moieties, particularly for the sulfone case. This contribution accounts for ∼90% of the transition in **D1** and has proportionally a much lower role in the excitation of the **D0** conformers. This decrease in the naphthalene-centered excitations is related to the larger involvement of the SO_*n*_ orbitals in the transition, corresponding to the electron lone pairs on the sulfur (**D0** and **D1**) and oxygen (**D1**) atoms, *i.e. n*(S) and *n*(SO) respectively. The presence of electron lone-pairs in the sulfide and sulfoxide linkers allows for sizeable CT_B_ contributions, related to the different electron density distributions found in the HOMOs (Fig. 3). CT_B_ contributions are already rather important in **D1** (15% in the lowest *syn* conformer) and become the main contribution in *syn*-**D0** (49%). On the other hand, the lack of available lone-pairs forbids the bridge-to-naphthalene electronic transitions in the S_1_ state of **D2**. For most of the cases, charge transfer between the two naphthalene fragments (CT) plays a minor role in the lowest excitation of the **Dn** dimers. It is worth noticing that the LE/CT electronic coupling increases with the number of oxygen atoms in the bridge. This trend can be attributed to the electronic screening by the sulfur electron lone-pairs, which limits the interactions between the two sets of π-electrons, as recently discussed in sulfur-bridged terthiophene dimers.^10^ On the other hand, the LE/CT_B_ couplings are much larger in the sulfide than in the sulfoxide bridge in accordance with the amount of CT_B_ in the excitation, which can be rationalized as a result of the presence of one additional electron lone-pair in the former.

### 3.3. Fluorescence emission

Thorough computational searches of the local minima on the lowest excited state PES of the naphthalene dimers identified a variety of states susceptible to decay back to the ground state *via* fluorescence emission (Table 3). The **Dn** dimers exhibit different structurally relaxed states corresponding to the stabilization of the π → π* excitations either localized on one naphthalene unit (L) or delocalized over both conjugated chromophores (D) or to the optimization of CT excitations from the SO_*n*_ bridge (for *n* = 0 and 1) to the π* naphthalene empty orbitals (CT_B_). The lack of sulfur lone-pairs prohibits the stabilization of bridge CT_B_ states on the S_1_ PES of **D2**, in line with the decomposition of the lowest excitation at the Franck–Condon (FC) geometry (Table 2).

**Table 3:** The vertical de-excitation energy Δ*E* (in eV), oscillator strength *f*, Stokes shift (in eV) and electronic character for the low-energy conformers of **D0**, **D1** and **D2** dimers computed at the ωB97X-D/6-31+G(d) level. The relative stabilities between the optimized excited states Δ*E*(rel) are also given (in kcal mol^–1^). The labels in the parenthesis represent the transitions involving π-type orbitals and indicate the localization on one naphthalene unit (L), delocalization over both naphthalene moieties (D) and the excimer state nature (E)

Dimer	Conf.	Character	Δ*E* ^*a*^ (em)	*f*	Δ*E* ^*b*^ (Stokes)	Δ*E*(rel)
**D0**	*syn*	π → π* (E)	3.12	0.110	1.12	6.7
	*syn*	*n*(S), π → π* (L)	3.58	0.316	0.66	1.9
	*anti*	*n*(S), π → π* (L)	3.39	0.215	0.85	2.6
	*syn*	*n*(S), π → π* (D)	3.51	0.325	0.73	2.1
	*anti*	*n*(S), π → π* (D)	3.43	0.322	0.81	0.0
**D1**	*syn*	π → π* (E)	2.95	0.078	1.45	0.0
	*syn*′	π → π* (E)	2.88	0.070	1.52	1.1
	*anti*	*n*(SO), π → π* (L)	3.65	0.191	0.76	3.7
	*perp*	*n*(SO), π → π* (L)	3.42	0.238	0.99	4.3
	*perp*′	*n*(SO), π → π* (L)	3.23	0.167	1.18	3.6
	*syn*	*n*(SO) → π* (D)	3.06	0.004	1.35	2.9
	*anti*	*n*(SO) → π* (D)	3.02	0.019	1.39	2.8
**D2**	*syn*	π → π* (E)	2.92	0.077	1.50	0.0
	*anti*	π → π* (L)	3.69	0.217	0.74	3.7
	*perp*	π → π* (L)	3.78	0.262	0.65	6.4

^*a*^The experimental emission maxima were obtained at 3.37 eV for the three dimers, while the measured Stokes shifts were 0.74, 0.82 and 0.79 eV for **D0**, **D1** and **D2**, respectively.^9^

^*b*^The computed Stokes shift with respect to the vertical absorption of the most stable ground state conformer.

The *syn*-**Dn** dimers hold excited state minima with excimeric nature and molecular geometries with the two naphthalene units close to the coplanar eclipsed relative orientation (Fig. S5†). These states present the largest Stokes shift for each dimer and are built as naphthalene π → π* excitations delocalized over the two chromophores without any involvement of the sulfur bridge. In addition, the large weights of the CT excitations (50% of the transition) for these states unequivocally identify them as naphthalene excimers (Fig. S6†). It is worth noting that while in **D0** the excimer is the energetically highest optimized excited state conformer, it is the most stable state in **D1** and **D2**, with larger interstate gaps in the latter. Moreover, the adiabatic energy gap with respect to the ground state *syn* conformer decreases as follows: **D0** > **D1** > **D2**, indicating a stronger excimer stabilization for the higher oxidation states of the bridge. The LE/CT couplings for the three *syn* excimers were computed at 626 (**D0**), 643 (**D1**) and 707 (**D2**) meV, considerably larger than the values obtained for the FC structures and with a trend in accordance with the electron lone-pairs screening of the electronic interactions.

The excited states with the highest oscillator strengths for the naphthalene dimers correspond to the π → π* excitations localized on one naphthalene or to the mixing between the π → π* and *n*(SO_*n*_) → π* (**D0** and **D1**) excitations. The computed vertical emission energies and Stokes shifts for these states are in very good agreement with the experimental measurements. The **D1** dimer also exhibits *syn* and *anti* low-lying states with virtually pure CT_B_ character and small oscillator strengths. The emission energies for the *n*(SO_*n*_), π → π* (L) states are in very good agreement with the photoluminescence frequencies and intensities computed using the model systems with only one naphthalene unit (Table S3†), confirming the localized nature of the transition.

**Fig. 4:**
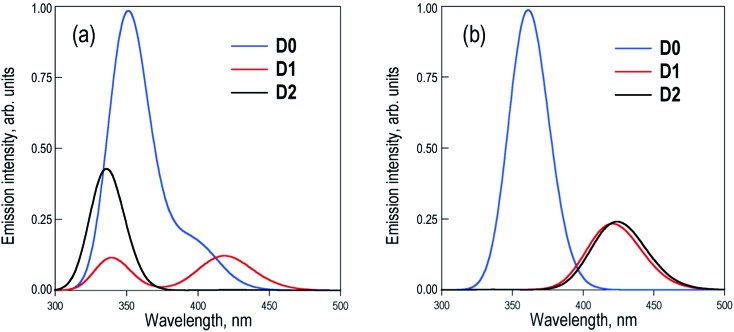
Simulation of the emission spectra of the **D0**, **D1** and **D2** dimers in DCM solution averaged over the (a) ground state and (b) excited state populations. Note that non-radiative decay was not considered in the simulations.

The excited state PES along the molecular torsion between the two naphthalene moieties of **Dn** dimers exhibit similar energy profiles to the ground state PES, with energy barriers for the conversion between the different conformers within the range of 2–7 kcal mol^–1^ (Fig. S2–S4†). The energy profiles of S_0_ and S_1_ PES around the ground state local minima are rather parallel, suggesting that depending on the experimental excitation conditions, two limiting situations might arise: (i) the initial excitation does not modify the conformer population and the emitting states are entirely controlled by the ground state equilibria or (b) the final emitting states are dictated by the relative stabilities between the minima in the S_1_ PES (excited state equilibria). The latter situation would be closer to the case with excitation energies high enough to surpass the torsion barriers. The simulated emission spectra recorded in DCM for the two limiting situations are shown in Fig. 4.

Estimation of the relative photoluminescence (PL) quantum yields obtained from the integration of the emission profiles (Table S5†), either considering ground or excited state Boltzmann populations (Fig. 4a and b respectively), indicates the **D0** dimer as the stronger emitter with a fluorescence efficiency three to four times larger than **D1** and **D2**. This result is in complete disagreement to the experimental observations, *i.e.* a much larger PL quantum yield (about one order of magnitude or more) in **D2** with respect to the **D0** and **D1** dimers.^9^ At this point, we must conclude that different state distributions over the computed S_1_ minima cannot account for the different PL efficiencies between the sulfur-bridged naphthalene dimers and that one or more non-radiative relaxation pathways (not considered so far) may play an important role in the deactivation of the **D0** and **D1** dimers. Furthermore, we explore the potential non-radiative S_1_ → S_0_ decays and rationalize how these mechanisms are favored in **D0** and **D1**, but not in **D2**, resulting in the much larger emission intensity found for the latter.

### 3.4. Non-radiative relaxation pathways

First, we consider the possibility of efficient internal conversion (IC) in the **D1** dimer following the pyramidal inversion mechanism on the excited state PES, as it has been proposed as a viable photo-induced process in aryl sulfoxides.^30^ The energy barriers computed for the inversion of the *syn* and *anti* **D1** conformers are 3.5 and 3.0 kcal mol^–1^, respectively, which are much lower than the energy required for the same structural rearrangement in the ground state (39 kcal mol^–1^). Hence, it seems that the inversion may be thermally available after photo-excitation of the sulfoxide dimer. However, for such a mechanism to result in an efficient IC to the ground state, a strong non-adiabatic coupling between the two states is required. Since the interstate couplings are inversely proportional to the energy gap, a small energy difference between the two PESs is necessary. Estimation of the S_0_/S_1_ energy gaps at the inversion TS were computed to be 32 and 50 kcal mol^–1^ for the *syn* and *anti* conformers, respectively (Table S6†). Hence, despite the availability of the photo-induced pyramidal inversion in **D1**, the magnitude of the S_0_/S_1_ gaps forced us to rule out the efficient non-radiative decay *via* IC at the inversion TS. Although the computed tetrahedral inversion barrier for **D2** in the lowest excited state is also considerably much lower than the ground state value, *i.e.* 41 *vs.* 97 kcal mol^–1^, the barrier is still too large to allow for photo-induced tetrahedral inversion. Furthermore, the S_0_/S_1_ energy gap at the TS is estimated at 29 kcal mol^–1^, blocking the non-radiative decay to the ground state *via* IC. Similar results have been obtained for the energy difference between the two states of **D0** at the inversion TS, *i.e.* 14 kcal mol^–1^ and 11 kcal mol^–1^ for the *syn*- and *anti*-conformers, respectively.

**Fig. 5:**
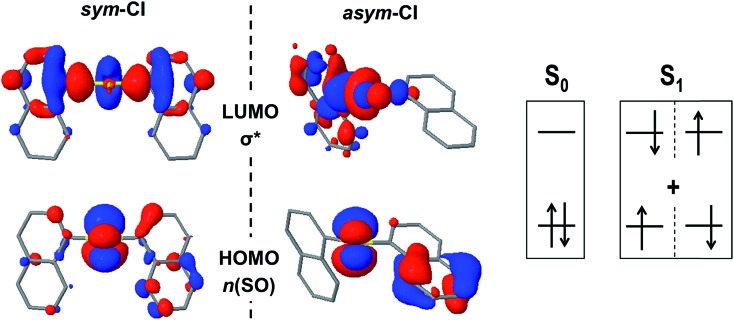
The frontier molecular orbitals *n*(SO) and σ* at the S_0_/S_1_ *sym*-CI and *asym*-CI points for the sulfoxide naphthalene dimer (**D1**).

In an attempt to find potential efficient non-radiative mechanisms for the photo-excited naphthalene dimers, we explore regions of the PES where the gap between the ground and lowest excited singlet state becomes small or where the two states become degenerate, that is S_0_/S_1_ intersections. For clarity, herein, we only discuss the results regarding the most stable conformations, that is, the *syn* (**D0** and **D1**) and *anti* (**D2**) forms. Motivated by the lowering of the S_0_/S_1_ gap at the TS of the pyramidal inversion for **D1**, we search for energy crossings from the trigonal planar arrangement of the SO_1_ bridge. Indeed, we identify a molecular geometry structurally related to the TS where the ground and excited PESs intersect with non-vanishing non-adiabatic couplings at the proximity of the crossing (ESI†), *i.e.* a conical intersection (CI). At this intersection, labelled as *sym*-CI, there is a symmetric elongation of the S–C bonds between SO_1_ and the naphthalene units and an important increase in the bridge C–S–C angle (Table 4). More importantly, the *sym*-CI point lies ∼0.66 eV below the S_1_ state at the FC region, and thus it is energetically accessible upon photo-excitation, providing a clear molecular mechanism to relax back to the ground state without photoemission. Similarly, we obtain a symmetric state crossing for the **D0** dimer, which exhibits a similar geometrical pattern (long C–S bonds and linear C–S–C angle). However, in this case, the *sym*-CI is obtained energetically above the FC S_1_ energy. For both dimers, **D0** and **D1**, at the *sym*-CI, the ground state crosses with the *n*(SO_*n*_) → σ* state, which is stabilized by the elongation of the two S–C bonds (Fig. 5). Moreover, in **D1**, the planarization of the sulfoxide group destabilizes the *n*(SO) due to π *anti*-bonding interactions with the p_z_ orbital of the oxygen atom. In the **D0** dimer, the *n*(S) destabilization comes from the interaction with the π-orbitals of the coplanar naphthalene fragments. On the other hand, the lack of electron lone-pairs in the sulfone bridge inhibits the presence of a low energy *sym*-CI in the **D2** dimer.

**Table 4:** The structural parameters (in Å and degrees) and relative energies (in eV) with respect to the S_1_ energy at the FC region of the inversion TS (*inv*-TS) and the *sym*-CI and *asym*-CI points for the *syn* conformers of the **D0** and **D1** dimers^*a*^

Dimer		State	*r*(C–S)	*α*(C–S–C)	Δ*E*(rel)
**D0**	*inv*-TS		1.80/1.80	178	+0.60
	*sym*-CI		2.17/2.12	178	+0.68
	*asym*-CI		2.26/1.77	176	–0.23
**D1**	*inv*-TS		1.76/1.76	115	+0.15
	*sym*-CI		1.96/1.88	155	–0.66
	*asym*-CI		2.32/1.78	107	–1.03

^*a*^The geometries for the *sym*-CI and *asym*-CI can be found in Fig. S7.

Excited state optimization within the CI subspace, *i.e.* minimal energy CI (MECI), of the sulfide and sulfoxide dimers results in non-symmetric molecular geometries (*asym*-CI) with one rather long S···C distance and a short S–C bond. As a result, σ* localizes on one side of the dimer (at the long S···C separation). This structural arrangement suggests a path towards molecular fragmentation. Furthermore, the computed *asym*-CI energies lie below the S_1_ energy in the FC region (and below the *sym*-CI point) and are therefore energetically available for both dimers. Hence, we identify the non-adiabatic relaxation of **D0** and **D1** dimers through *asym*-CI as the mechanism describing reversible molecular fragmentation (although the molecule has not been effectively fragmented in *asym*-CI), where there is an elongation and shrinking of a S–C bond resulting in a fast decay to the electronic ground state. The reverse fragmentation mechanism has been proposed as the main inversion of aryl sulfoxides bearing a primary alkyl group.^29,31,32,34^ Moreover, we find that such a mechanism can be potentially photoinduced in the sulfide and sulfoxide aromatic dimers and proceeds through a MECI.

**Fig. 6:**
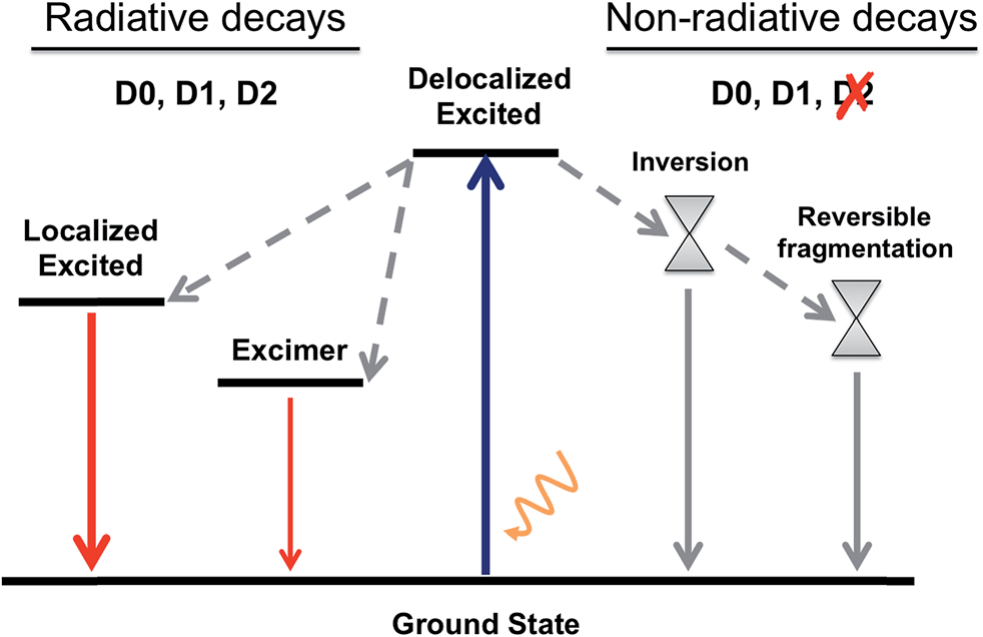
A general Jablonski diagram for the deactivation mechanisms after photoexcitation of the **D0**, **D1** and **D2** naphthalene dimers.

By gathering the results discussed above regarding the photoexcitation and different deactivation paths of the naphthalene dimers studied, it is possible to draw a general picture for the photophysical properties of **Dn**. The main photophysical mechanisms explored are represented in the Jablonski diagram in Fig. 6. Relaxation of the photoexcited sulfur-bridged naphthalene dimers allows the formation of strongly emissive localized excitations and weakly emitting excimers. Moreover, sulfide and sulfoxide dimers exhibit non-radiative decay back to the ground state, which actually dominate their excited state dynamics in solution. On the other hand, the lack of electron lone-pairs in **D2** blocks the presence of low-lying ground-excited state crossings, resulting in much larger PL yields with respect to **D0** and **D1**.

## 4. Conclusions

In this study we have identified and characterized both the radiative and non-radiative deactivation mechanisms occurring in sulfur-bridged naphthalene dimers. The different PL efficiencies upon oxidation of the sulfur-bridge have been rationalized by the existence of energetically available non-radiative decays for the sulfide and sulfoxide bridges. The lack of electron lone-pairs in the sulfone linker is the origin of the much stronger PL with respect to the S and SO_1_ cases.

Although the computed vertical transition energies and their intensities in the FC structures are very similar for the three naphthalene dimers, there are significant differences in the character of the transition to the lowest excited singlet between **D0**, **D1** and **D2**, which is the larger involvement of the *n*(SO_*n*_) orbitals in terms of bridge → naphthalene CT with lower oxidation state of the sulfur atom. Geometrical relaxation to the local minima of the excited state PESs cannot account for the very weak PL of **D0** and **D1**, pointing towards the existence of efficient non-radiative decays, not present from the excited **D2**. We identify energy crossing regions in **D0** and **D1** dimers that allow the conversion of the photo-excited molecules back to the ground state with no fluorescence emission. Our calculations indicate that while two types of S_0_/S_1_ state crossings, *i.e.* symmetric and asymmetric, may be reached along the excited state decay of **D1**, only the asymmetric intersection is energetically available for the **D0** dimer. The identification of energetically available asymmetric CI pointing towards the reversible molecular fragmentation suggests a photoinduced roaming mechanism^35^ as a potential non-radiative deactivation path of **D0** and **D1**. Finally, it is important to notice that in our calculations, due to the nature of the studied chromophores, we have not considered the role of ISC as one of the main deactivation channels.

The present results suggest that, differently to the terthiophene dimers, the SO_*n*_ bridged naphthalene bichromophores do not require efficient ISC to limit the fluorescence emission. On the other hand, our results reinforce the generality of the electron lone-pair screening concept for sulfur-bridged chromophore dimers. The obtained results and conclusions are general enough to be extrapolated to other sulfur-bridged conjugated dimers, therefore proportionating novel strategies for the design of strongly luminescent organic molecules with controlled charge transfer. Investigations in this direction are currently underway in our laboratories.
