# Supplementary material for: The photophysics of naphthalene dimers controlled by sulfur bridge oxidation
Source: Chem Sci. 2017 Apr 24;8(7):4941–50. doi: 10.1039/c7sc01285c (PMC5607855; doi:10.1039/c7sc01285c)
Supplement: Supplementary file 1 [file SC-008-C7SC01285C-s001.pdf]

Electronic Supplementary Material (ESI) for Chemical Science.
This journal is © The Royal Society of Chemistry 2017

Supporting Information

Photophysics of Naphthalene Dimers Controlled by the Sulfur Bridge Oxidation

Clàudia Climent[a], Mario Barbatti[b], Michael O. Wolf[c], Christopher J. Bardeen[d], David Casanova[e,f,]*

[a]Departament de Ciència de Materials i Química Física and Institut de Química Teòrica i Computacional (IQTCUB), Universitat de Barcelona, Martí i Franquès 1-11, Barcelona 08028, Spain.
[b]Aix Marseille Univ, CNRS, ICR, Marseille, France.
[c]Department of Chemistry, University of British Columbia, 2036 Main Mall, Vancouver, BC, V6T 1Z1, Canada.
[d]Department of Chemistry, University of California Riverside, 501 Big Springs Road, Riverside, California 92521, United States.
[e]Kimika Facultatea, Euskal Herriko Unibertsitatea (UPV/EHU), Donostia International Physics Center, Paseo Manuel de Lardizabal, 4, Donostia 20018, Spain.
[f]IKERBASQUE, Basque Foundation for Science, Bilbao 48013, Spain.

Table of Contents

TABLES

Table S1. Vertical transition energies to the lowest excited bright singlet state for the *anti*-**D2** dimer computed with different functionals and conditions.

Table S2. Vertical transition energies to the lowest excited bright singlet state for the *anti*-**D2** dimer computed at the TDDFT ωB97X-D level with different basis

Table S3. Vertical transition energies ΔE (in eV) and oscillator strengths (*f*) for the naphthalene-SO_n-Me molecules in DCM computed at the ωB97X-D/6-31+G(d) level.

Table S4. Vertical transition energies ΔE (in eV), oscillator strength (*f*), electronic character (in %) LE (on naphthalene fragments), CT (between naphthalene moieties) and CT_B (from the SO_n bridge to the naphthalenes) and electronic couplings between the lowest LE, CT and CT_B diabatic states (in meV) for the lowest excited singlet of the different conformers of **D0**, **D1** and **D2** dimers in DCM computed at the ωB97X-D/6-31+G(d) level.

Table S5. Estimated relative PL quantum yields (without considering non-radiative decay pathways) obtained as the numerical integration of the emission profiles of **Dn** dimers in DCM considering (i) ground state population (S_0) and (ii) excited state population (S_1) compared to the experimental yields from J. Amer. Chem. Soc, *135* (**2013**) 8109.

Table S6. S1/S0 energy gaps (in kcal/mol) at the inversion TS of **D1** (*syn* and *anti*) and **D2** (*anti*) dimers estimated as: $\Delta E(S_1/S_0) = \Delta E(S_0 \rightarrow S_1) - \lambda(S_1) + \Delta E(TS,S_1) - \Delta E(TS,S_0)$, where $\Delta E(S_0 \rightarrow S_1)$ is the vertical gap at the Franck-Condon geometry, $\lambda(S_1)$ is the reorganization energy of S_1, and $\Delta E(TS,S_0)$ and $\Delta E(TS,S_1)$ are the energy barriers at the S_0 transition state geometry on the S_0 and S_1 PES, respectively.

FIGURES

Figure S1. Most stable conformers for the ground state of **Dn** dimers in DCM solution.

Figure S2. Ground and first excited state energy profiles for the interconversion between conformers of the **D0** dimer in DCM solution computed at the ωB97X-D/6-31+G(d) level. All structures have been relaxed on the ground state PES.

Figure S3. Ground and first excited state energy profiles for the interconversion between conformers of the **D1** dimer in DCM solution computed at the ωB97X-D/6-31+G(d) level. All structures have been relaxed on the ground state PES.

Figure S4. Ground and first excited state energy profiles for the interconversion between conformers of the **D2** dimer in DCM solution computed at the ωB97X-D/6-31+G(d) level. All structures have been relaxed on the ground state PES.

Figure S5. Molecular structure of excimeric states for **Dn** dimers optimized at the ωB97X-D/6-31+G(d) level in DCM solution.

Figure S6. HOMO and LUMO orbitals accounting for the main electronic excitation contribution of **Dn** excimers computed at the ωB97X-D/6-31+G(d) level in DCM solution.

Figure S7. Molecular structures of **D0** (top) and **D1** (bottom) dimers at the *sym*-CI and *asym*-CI for the **Dn** dimers

COMPUTATIONAL DETAILS

Transition states for the structural inversion.

CI relative energies.

Derivative couplings.

OPTIMIZED GEOMETRIES

Ground state optimized coordinates for the Dn dimers.

Transition state structures for the inversion of Dn dimers.

TABLES

Table S1. Vertical transition energies to the lowest excited bright singlet state for the *anti*-**D2** dimer computed with different functionals and conditions.

	TDDFT ωB97XD vacuum	TDA ωB97XD vacuum	TDDFT CAM-B3LYP vacuum	TDDFT ωB97XD DCM	experimental
bright S_n	S_1	S_3	S_1	S_1	-
eV	4.56	4.78	4.55	4.50	4.16
Δexp / eV	0.40	0.62	0.39	0.34	-
nm	272	259	273	276	298
f	0.20	0.20	0.19	0.26	-

All values correspond to the 6-31G(d) basis set for the ωB97X-D/6-31G(d) optimized geometry in vacuum.

Table S2. Vertical transition energies to the lowest excited bright singlet state for the *anti*-**D2** dimer computed at the TDDFT ωB97X-D level with different basis sets.

basis set	6-31G(d)	6-31+G(d)	experimental
bright S_n	S_1	S_1	-
eV	4.50	4.42	4.16
Δexp / eV	0.34	0.26	-
nm	276	281	298
f	0.26	0.27	-

Table S3. Vertical transition energies ΔE (in eV) and oscillator strengths (*f*) for the naphthalene-SO_n-Me molecules in DCM computed at the ωB97X-D/6-31+G(d) level.

SO_n	geometry	ΔE	*f*
**S**	S_0	4.53	0.18
	S_1	3.50	0.23
**SO_1**	S_0	4.52	0.19
	S_1	3.73	0.20
**SO_2**	S_0	4.52	0.18
	S_1	3.81	0.21

Table S4. Vertical transition energies ΔE (in eV), oscillator strength (*f*), electronic character (in %) LE (on naphthalene fragments), CT (between naphthalene moieties) and CT_B (from the SO_n bridge to the naphthalenes) and electronic couplings between the lowest LE, CT and CT_B diabatic states (in meV) for the lowest excited singlet of the different conformers of **D0**, **D1** and **D2** dimers in DCM computed at the ωB97X-D/6-31+G(d) level.

dimer	*conf.*	ΔE^a	*f*	LE	CT	CT_B	LE/CT	LE/CT_B
**D0**	*syn*	4.24	0.335	37	14	49	102	509
	anti	4.31	0.326	59	5	36	65	244
	anti'	4.37	0.347	68	9	23	119	149
**D1**	*syn*	4.41	0.301	84	1	15	129	200
	syn'	4.35	0.280	78	4	18	98	221
	anti	4.43	0.312	91	1	8	126	195
	perp	4.33	0.329	44	11	45	128	153
**D2**	*syn*	4.38	0.273	94	6	0	121	0
	anti	4.42	0.272	96	4	0	162	0
	perp	4.47	0.341	100	0	0	41	0

[a]Experimental absorption maxima: 4.11 eV (**D0**), 4.19 eV (**D1**) and 4.16 eV (**D2**) [J. Am. Chem. Soc, *135* (**2013**) 8109].

Table S5. Estimated relative PL quantum yields (without considering non-radiative decay pathways) obtained as the numerical integration of the emission profiles of **Dn** dimers in DCM considering (i) ground state population (S_0) and (ii) excited state population (S_1) compared to the experimental yields from J. Amer. Chem. Soc, *135* (**2013**) 8109.

dimer	PL(S_0)	PL(S_1)	PL(exp)
**D0**	1.00	1.00	1.00
**D1**	0.22	0.32	2.92
**D2**	0.31	0.33	22.50

Table S6. S1/S0 energy gaps (in kcal/mol) at the inversion TS of **D1** (*syn* and *anti*) and **D2** (*anti*) dimers estimated as: $\Delta E(S_1/S_0) = \Delta E(S_0 \rightarrow S_1) - \lambda(S_1) + \Delta E(TS,S_1) - \Delta E(TS,S_0)$, where $\Delta E(S_0 \rightarrow S_1)$ is the vertical gap at the Franck-Condon geometry, $\lambda(S_1)$ is the reorganization energy of S_1, and $\Delta E(TS,S_0)$ and $\Delta E(TS,S_1)$ are the energy barriers at the S_0 transition state geometry on the S_0 and S_1 PES, respectively.

dimer	conformer	$\Delta E(S_1/S_0)$
**D1**	*syn*	32.4
	anti	49.5
**D2**	*anti*	29.1

FIGURES

Figure S1. Most stable conformers for the ground state of **D0** (top) and **D2** (bottom) dimers in DCM solution.

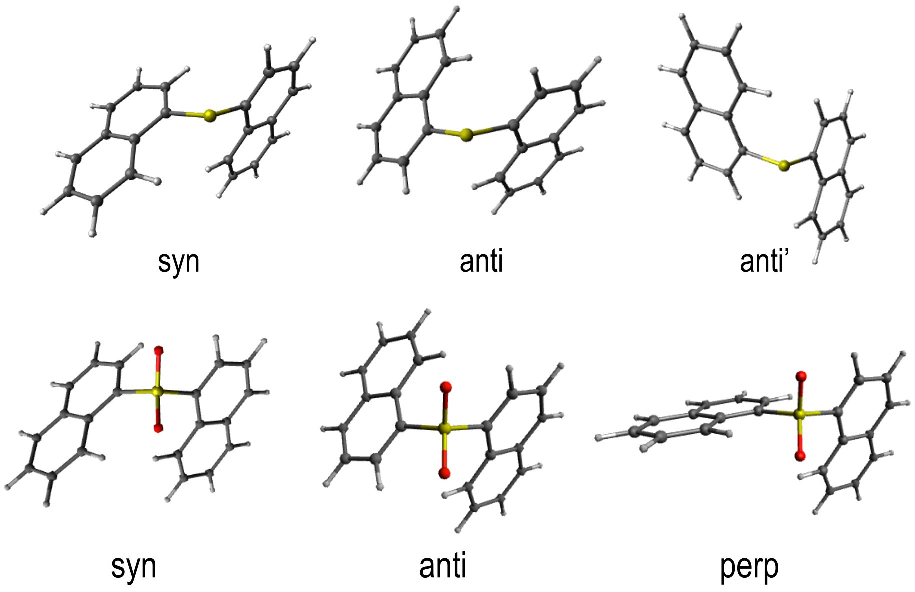

Figure S2. Ground and first excited state energy profiles for the interconversion between conformers of the **D0** dimer in DCM solution computed at the ωB97X-D/6-31+G(d) level. All structures have been relaxed on the ground state PES.

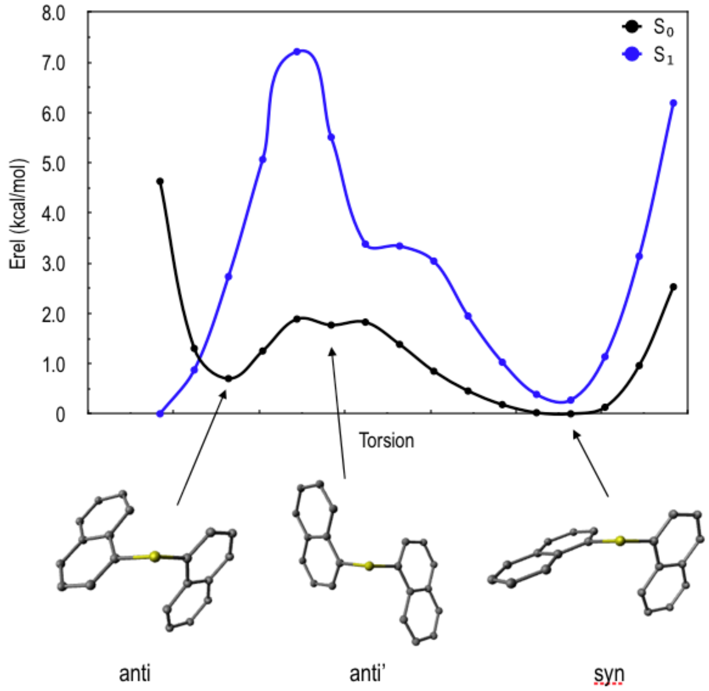

Figure S3. Ground and first excited state energy profiles for the interconversion between conformers of the **D1** dimer in DCM solution computed at the ωB97X-D/6-31+G(d) level. All structures have been relaxed on the ground state PES.

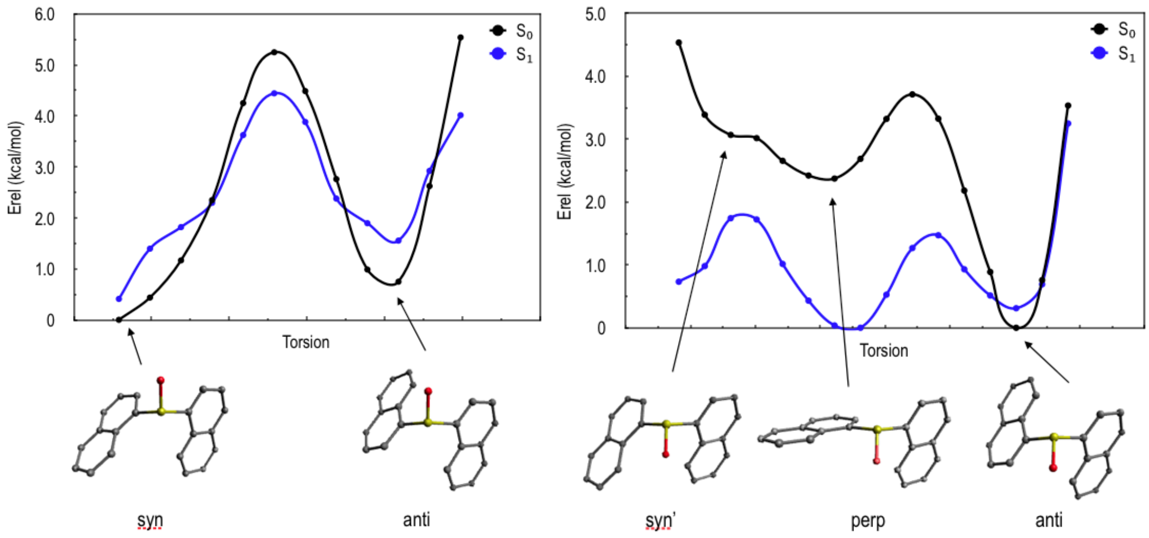

Figure S4. Ground and first excited state energy profiles for the interconversion between conformers of the **D2** dimer in DCM solution computed at the ωB97X-D/6-31+G(d) level. All structures have been relaxed on the ground state PES

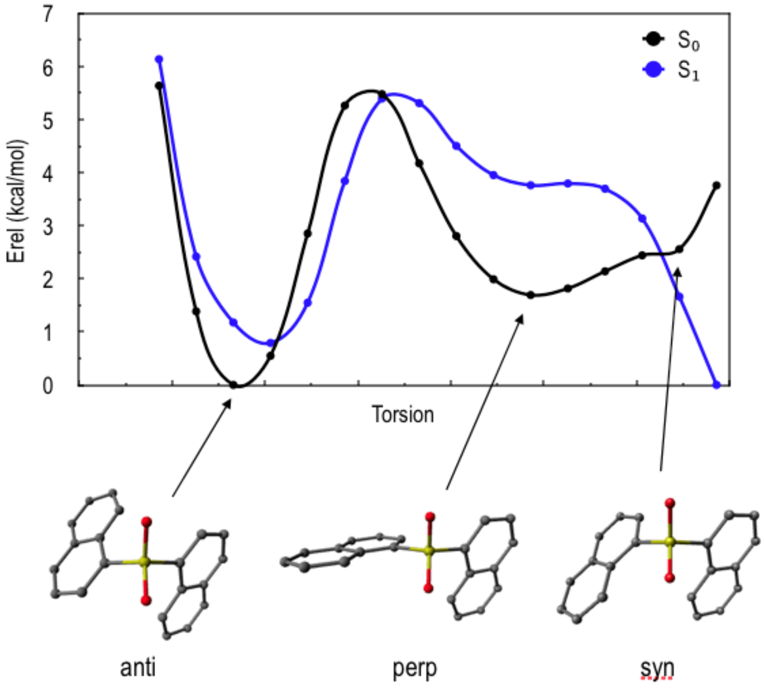

Figure S5. Molecular structure of excimeric states for **Dn** dimers optimized at the ωB97X-D/6-31+G(d) level in DCM solution.

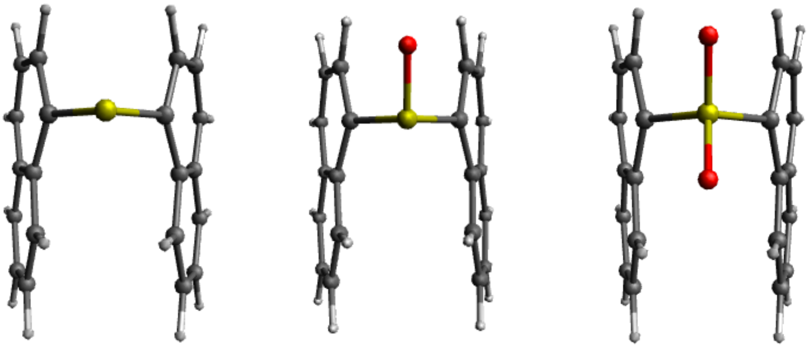

D0 **D1** **D2**

Figure S6. HOMO and LUMO orbitals accounting for the main electronic excitation contribution of **Dn** excimers computed at the ωB97X-D/6-31+G(d) level in DCM solution.

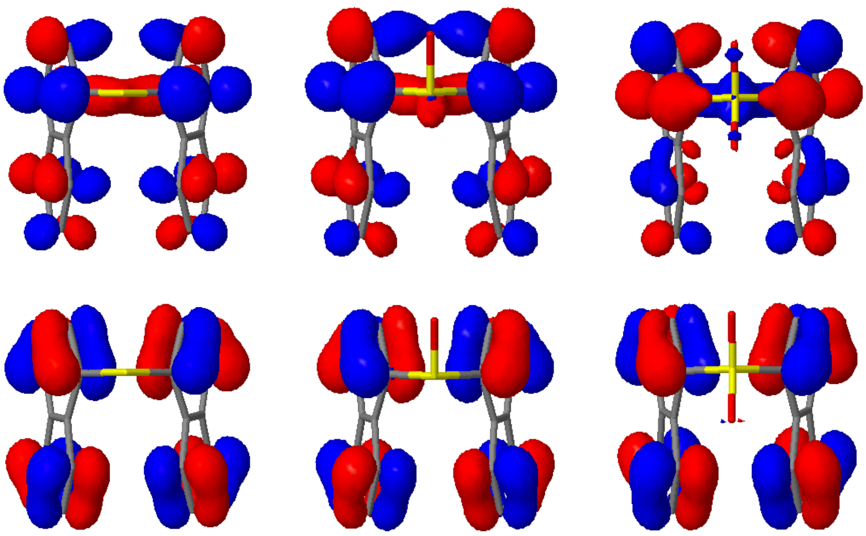

D0 **D1** **D2**

Figure S7. Molecular structures of **D0** (top) and **D1** (bottom) dimers at the *sym*-CI and *asym*-CI for the **Dn** dimers.

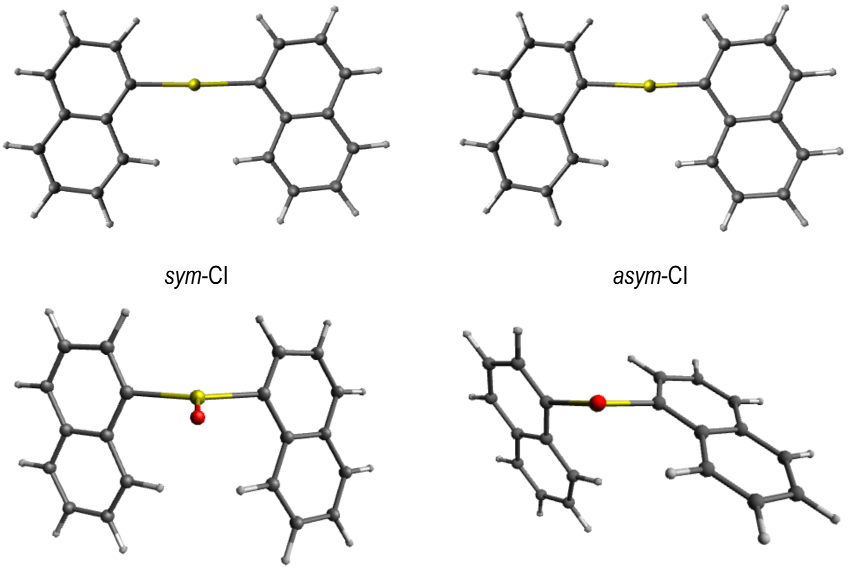

COMPUTATIONAL DETAILS

Transition states for the structural inversion

The transition states for the structural inversion of **D1** and **D2** were characterized as first order saddle points while those of **D0** were second order saddle points on the ground state PES. This second order saddle point which corresponds to a linear structure of **D0** is a transition state connecting two transition states (first order saddle points). These latter transition states connect the two enantiomers of *syn* and *anti* ground state conformers respectively.

CI relative energies

The values of ΔE(rel) for *inv*-TS and *sym*-CI (Table 4 of the main text) were calculated within the TDDFT. The values of ΔE(rel) for *asym*-CI were calculated by the following expression, since TDDFT cannot properly describe the situation of bond fragmentation.

$\Delta E(\mathrm{rel}) = \Delta sym + \Delta asym$

$\Delta sym = \Delta E(\mathrm{rel})$ for *sym*-CI

$\Delta asym$ = relative energy of *asym*-CI with respect to *sym*-CI calculated by SF-DFT

Derivative couplings

Derivative couplings (atomic units) for the **D0** and **D1** dimers.

sym-CI	**D0**	71.3
	**D1**	169.8
asym-CI	**D0**	5550.8
	**D1**	2644.5

Note. The derivative coupling vectors correspond to the same nuclear motion as the imaginary frequencies of the transition states of **D1** and **D0**. Therefore, the nuclear motion that lowers the energy from the transition states to the ground state minima is equivalent to the motion that opens the gap of the S_0/S_1 crossing.

OPTIMIZED GEOMETRIES

Ground state optimized coordinates for the Dn dimers (in Angstroms).

syn-**D0** coordinates

6	-4.380813	0.053742	-0.139065
6	-3.102629	-0.349201	-0.613288
6	-2.656491	0.010328	-1.912501
6	-2.255339	-1.118771	0.239365
6	-1.419179	-0.382123	-2.353735
6	-0.578337	-1.160650	-1.526894
6	-0.974816	-1.521097	-0.259709
6	4.328485	-1.290494	-0.433799
6	3.975599	1.005679	0.417735
6	3.498800	-0.311664	0.179157
6	3.177877	1.944966	1.021386
6	2.171749	-0.654604	0.571714
6	3.864098	-2.559237	-0.660346
6	1.709533	-1.984332	0.299775
6	1.374302	0.338101	1.202094
6	2.539461	-2.903708	-0.299466
6	1.864544	1.602517	1.420506
1	1.238885	2.346342	1.905175
1	4.499615	-3.304499	-1.128759
1	0.365932	0.090673	1.517318
1	2.170602	-3.903737	-0.506555
16	0.080159	-2.522327	0.787337
1	-1.076033	-0.106695	-3.346574
6	-2.721913	-1.440386	1.544383
6	-4.805301	-0.281254	1.121812
1	-4.296428	-1.288373	2.974361
6	-3.961837	-1.033918	1.972894
1	-2.086149	-2.009517	2.215223
1	0.393170	-1.469882	-1.900947
1	5.340189	-1.014084	-0.719707
1	4.988556	1.256299	0.112667
1	3.551780	2.949146	1.198018
1	-3.309819	0.601820	-2.548600
1	-5.018715	0.637732	-0.797937
1	-5.784110	0.033296	1.471783

anti-**D0** coordinates

6	-3.605101	-0.734785	-0.065864
6	-2.938248	0.507646	-0.244064
6	-3.265721	1.359270	-1.333103
6	-1.945786	0.910149	0.699896
6	-2.635975	2.566223	-1.488163
6	-1.623512	2.956526	-0.582033
6	-1.269676	2.153803	0.479486
6	2.907644	0.022612	0.590775
6	3.567318	-0.481284	-0.562975
6	3.239441	-0.457408	1.886191
6	2.616123	0.046800	2.997305
6	1.918031	1.040786	0.441754
6	1.606222	1.026110	2.857849
6	1.248714	1.509087	1.618747
16	-0.007796	2.786476	1.583620
6	-1.692892	0.061876	1.811997
6	-3.321329	-1.540630	1.007539
1	-2.153000	-1.758301	2.821420
6	-2.361073	-1.128780	1.961110
1	2.874907	-0.314237	3.988053

6	3.279330	0.009150	-1.811424
6	1.661062	1.541874	-0.863356
6	2.322218	1.040369	-1.957739
1	1.090075	1.387737	3.742852
1	-2.891492	3.220832	-2.315850
1	-1.102055	3.896546	-0.739638
1	-0.967901	0.363783	2.557776
1	0.938257	2.337387	-0.995755
1	2.110977	1.442031	-2.944532
1	4.001292	-1.226922	1.980895
1	3.790410	-0.382873	-2.685803
1	4.312741	-1.262428	-0.435930
1	-3.838025	-2.487410	1.134127
1	-4.352742	-1.032755	-0.796743
1	-4.029569	1.039271	-2.037188

syn-**D1** coordinates

6	3.774638	0.207290	0.385030
6	3.911179	-0.800359	-1.874257
6	3.211269	-0.042437	-0.894805
6	3.364513	-1.031039	-3.109573
6	1.921368	0.481927	-1.199355
6	3.094520	0.943696	1.322651
6	1.244383	1.245208	-0.210128
6	1.384439	0.197741	-2.490146
6	1.817480	1.469703	1.017775
6	2.081869	-0.520830	-3.423738
1	1.637049	-0.695754	-4.399099
1	3.906893	-1.605306	-3.854133
1	1.285720	2.056969	1.760388
1	0.265572	1.666660	-0.416962
6	-2.848496	-1.926222	-0.393552
6	-4.034346	0.020563	0.567966
6	-3.046069	-0.524275	-0.296855
6	-4.225736	1.375658	0.652125
6	-2.244608	0.356966	-1.083536
6	-1.895702	-2.451215	-1.230967
6	-1.252061	-0.229237	-1.923682
6	-2.472669	1.758155	-0.976906
6	-1.086328	-1.590257	-2.001729
6	-3.436151	2.250395	-0.132597
1	-3.596040	3.322492	-0.066901
1	-1.752964	-3.524898	-1.300177
1	-1.883383	2.448803	-1.571866
1	-0.326358	-2.009430	-2.655479
16	-0.236872	0.839224	-2.981064
8	-0.449885	0.388245	-4.414458
1	3.534368	1.127842	2.298136
1	4.758614	-0.196562	0.609398
1	4.894391	-1.191563	-1.626180
1	-3.469074	-2.583103	0.210334
1	-4.982665	1.782044	1.316042
1	-4.637866	-0.660220	1.162829

anti-**D1** coordinates

6	3.599130	-0.727714	0.430856
6	2.708226	-1.858474	-1.578387
6	2.757526	-0.730825	-0.713464
6	1.916416	-1.857406	-2.696254
6	1.964265	0.419383	-1.000755
6	3.667361	0.371037	1.249810
6	2.077877	1.550550	-0.149206
6	1.111499	0.361488	-2.147045

6	2.904606	1.522810	0.947429
6	1.111513	-0.730810	-2.981554
1	0.475833	-0.740501	-3.863551
1	1.895013	-2.713987	-3.362157
6	-2.683489	2.082037	1.098111
6	-3.613027	-0.189430	0.790881
6	-2.750780	0.860278	0.374716
6	-3.702685	-1.355215	0.073040
6	-1.956578	0.686832	-0.798032
6	-1.873938	3.104107	0.676718
6	-1.088112	1.758096	-1.168217
6	-2.087137	-0.525319	-1.528878
6	-1.069498	2.939136	-0.474459
6	-2.937083	-1.517210	-1.104921
1	-1.837967	4.041605	1.222727
1	-0.420579	3.741948	-0.812268
16	-0.034915	1.664474	-2.650428
8	0.702834	2.988870	-2.715093
1	-4.207859	-0.048813	1.689760
1	-4.366261	-2.150287	0.399381
1	-3.297661	2.196668	1.987486
1	-3.024513	-2.432998	-1.681928
1	-1.522819	-0.673373	-2.442181
1	4.193527	-1.612648	0.643175
1	3.322327	-2.723449	-1.341492
1	4.314100	0.364790	2.122019
1	2.979897	2.399029	1.584618
1	1.528720	2.452964	-0.383159

syn-**D2** coordinates

6	-3.951095	-1.107175	0.250677
6	-3.037357	-0.116837	-0.202130
6	-3.009143	0.250600	-1.572030
6	-2.151901	0.507882	0.732166
6	-2.141298	1.207147	-2.030695
6	-1.255287	1.828666	-1.129399
6	-1.252140	1.489130	0.204793
6	3.914456	1.533694	-1.003253
6	3.775561	-0.878509	-0.490916
6	3.243141	0.435067	-0.400301
6	3.128384	-1.941069	0.086899
6	2.021219	0.648601	0.304007
6	3.404095	2.802978	-0.930780
6	1.521991	1.988545	0.342743
6	1.381608	-0.470966	0.902369
6	2.184027	3.031229	-0.255475
6	1.920683	-1.729358	0.790316
1	1.414199	-2.570346	1.254351
1	3.921664	3.636527	-1.393937
1	0.467193	-0.341708	1.468167
1	1.763067	4.030016	-0.211195
16	-0.019736	2.388880	1.164789
1	-2.124516	1.488888	-3.078168
6	-2.235141	0.111044	2.097960
6	-3.998707	-1.471777	1.570595
1	-3.172557	-1.134477	3.544904
6	-3.130522	-0.850432	2.497477
1	-1.586546	0.574805	2.828158
1	-0.564618	2.578965	-1.503088
8	-0.270287	3.819909	0.961786
8	0.046889	1.893962	2.543685
1	4.846861	1.347065	-1.529592
1	3.541301	-2.942266	0.010718

1	4.707772	-1.026015	-1.029793
1	-3.694138	-0.242182	-2.256835
1	-4.614637	-1.569612	-0.475361
1	-4.699923	-2.229249	1.907212

anti-**D2** coordinates

6	-3.641774	-0.863254	0.260802
6	-2.806288	0.130195	2.359144
6	-2.808382	0.065333	0.939673
6	-2.013195	1.026213	3.026077
6	-1.976624	0.945563	0.184275
6	-3.663385	-0.918564	-1.109293
6	-2.040049	0.878040	-1.234212
6	-1.138957	1.838835	0.925009
6	-2.857012	-0.032148	-1.859055
6	-1.161085	1.883926	2.297187
1	-0.511862	2.576961	2.821769
1	-2.021378	1.076866	4.109793
1	-1.456567	1.561428	-1.836868
6	2.804250	0.316977	-2.362095
6	3.631038	-0.862314	-0.358569
6	2.805646	0.128879	-0.953641
6	3.652870	-1.035794	1.001630
6	1.981363	0.947212	-0.124066
6	2.018280	1.273748	-2.948203
6	1.149898	1.907180	-0.783934
6	2.045440	0.757347	1.283206
6	1.172289	2.070955	-2.147063
6	2.855103	-0.210125	1.826166
1	2.887823	-0.335653	2.904256
1	2.027053	1.418375	-4.023416
1	1.468639	1.391378	1.943492
1	0.527465	2.811197	-2.608891
16	0.008133	2.955926	0.117484
8	0.750806	3.664997	1.166085
8	-0.731467	3.755551	-0.866057
1	3.446171	-0.314040	-2.971048
1	4.249003	-1.480399	-1.004510
1	4.286264	-1.796133	1.448120
1	-3.453172	-0.546626	2.911081
1	-2.888917	-0.064351	-2.943970
1	-4.302939	-1.631873	-1.619990
1	-4.266310	-1.528790	0.850803

Transition state structures for the inversion of Dn dimers (in Angstroms).

Saddle point for the inversion of *syn*-**D0**

6	-4.834903	-1.904762	2.258995
6	-3.928285	-2.123741	1.185933
6	-4.384386	-2.200049	-0.157011
6	-2.538023	-2.268200	1.482107
6	-3.499575	-2.414266	-1.181717
6	-2.116324	-2.558917	-0.923584
6	-1.667731	-2.481504	0.370765
6	4.605981	-3.202335	1.320169
6	4.650106	-1.279935	2.879115
6	3.942738	-2.148613	2.003680
6	4.010092	-0.260093	3.536442
6	2.540934	-1.942794	1.819348
6	3.912035	-4.030467	0.476697
6	1.874573	-2.843258	0.934400
6	1.907563	-0.877162	2.507969
6	2.523075	-3.855848	0.273657

6	2.622818	-0.057270	3.345145
1	2.120518	0.754389	3.862984
1	4.417605	-4.835461	-0.047858
1	0.844936	-0.718434	2.355480
1	1.979136	-4.515248	-0.391379
16	0.100568	-2.646706	0.680782
1	-3.846554	-2.474395	-2.208739
6	-2.113399	-2.196612	2.833232
6	-4.394980	-1.834920	3.556414
1	-2.673699	-1.934701	4.873035
6	-3.017721	-1.985183	3.844269
1	-1.057740	-2.318146	3.053496
1	-1.421142	-2.724558	-1.737362
1	5.672296	-3.340124	1.476124
1	5.716152	-1.441283	3.017076
1	4.565015	0.394681	4.201680
1	-5.446085	-2.087070	-0.358012
1	-5.891320	-1.793755	2.028316
1	-5.100555	-1.667210	4.364647

TS inversion for *syn*-**D1**

6	1.474437	0.083210	-2.504752
6	2.258714	-0.313616	-3.564971
6	3.642390	-0.523303	-3.381661
6	4.199180	-0.380179	-2.136257
6	3.410769	0.018143	-1.023554
6	3.991318	0.177542	0.263827
6	3.238730	0.604454	1.328151
6	1.868481	0.905297	1.146578
6	1.274175	0.754897	-0.082095
6	2.021921	0.294605	-1.198135
1	1.811690	-0.470033	-4.541346
1	4.250710	-0.824508	-4.228352
1	5.258872	-0.566851	-1.984093
1	5.048623	-0.041677	0.387815
1	3.692447	0.723078	2.307391
1	1.280666	1.263671	1.986296
1	0.224377	1.000061	-0.199648
6	-3.003324	-1.851131	-0.001558
6	-1.991078	-2.506836	-0.654685
6	-1.172832	-1.813985	-1.574835
6	-1.361625	-0.465621	-1.777206
6	-2.391257	0.262897	-1.103687
6	-2.598461	1.657906	-1.264376
6	-3.602948	2.295702	-0.577884
6	-4.450840	1.574320	0.295356
6	-4.267507	0.225895	0.473198
6	-3.232415	-0.464325	-0.212634
1	-3.645681	-2.386625	0.692303
1	-1.822802	-3.566195	-0.489898
1	-0.395148	-2.344661	-2.114653
1	-1.965220	2.217967	-1.946097
1	-3.749899	3.363579	-0.708596
1	-5.243890	2.092505	0.825950
1	-4.909645	-0.334561	1.147697
16	-0.233776	0.378469	-2.833561
8	-0.698161	1.316783	-3.975499

TS inversion for **D2**

6	-4.683281	1.324949	1.640483
6	-3.956242	3.500046	2.564416
6	-3.729938	2.376568	1.724986
6	-3.020439	4.495095	2.670996

6	-2.530072	2.292874	0.955287
6	-4.462497	0.233661	0.840466
6	-2.333438	1.146765	0.136187
6	-1.587239	3.353175	1.101682
6	-3.270458	0.146016	0.082614
6	-1.809201	4.411758	1.945142
1	-1.065468	5.193105	2.061525
1	-3.193476	5.350467	3.316623
1	-1.436442	1.058236	-0.467318
6	3.979188	3.687067	-2.272767
6	4.691098	1.435659	-1.539119
6	3.743988	2.496202	-1.534800
6	4.460615	0.279589	-0.839035
6	2.540936	2.352650	-0.779143
6	3.048905	4.692715	-2.295529
6	1.605344	3.427712	-0.834009
6	2.333825	1.139972	-0.065103
6	1.835194	4.553986	-1.582176
6	3.264611	0.132480	-0.096706
1	3.082841	-0.784733	0.455387
1	3.228454	5.599757	-2.864320
1	1.432625	1.003533	0.523482
1	1.096033	5.346734	-1.631574
16	0.009105	3.358689	0.132515
8	0.798553	3.301192	1.422103
8	-0.779780	3.402512	-1.157964
1	4.908875	3.783223	-2.827227
1	5.604343	1.558830	-2.115710
1	5.190435	-0.524282	-0.852148
1	-4.883873	3.552909	3.128075
1	-3.096725	-0.719487	-0.549716
1	-5.197120	-0.564063	0.785827
1	-5.593506	1.401873	2.229773
